# Supplementary material for: Human-Induced Neural and Mesenchymal Stem Cell Therapy Combined with a Curcumin Nanoconjugate as a Spinal Cord Injury Treatment
Source: Int J Mol Sci. 2021 May 31;22(11):5966. doi: 10.3390/ijms22115966 (PMC8198521; doi:10.3390/ijms22115966)
Supplement: Supplementary file 1 [file ijms-22-05966-s001.zip › ijms-1229181-supplementary.pdf]

Supplementary Data

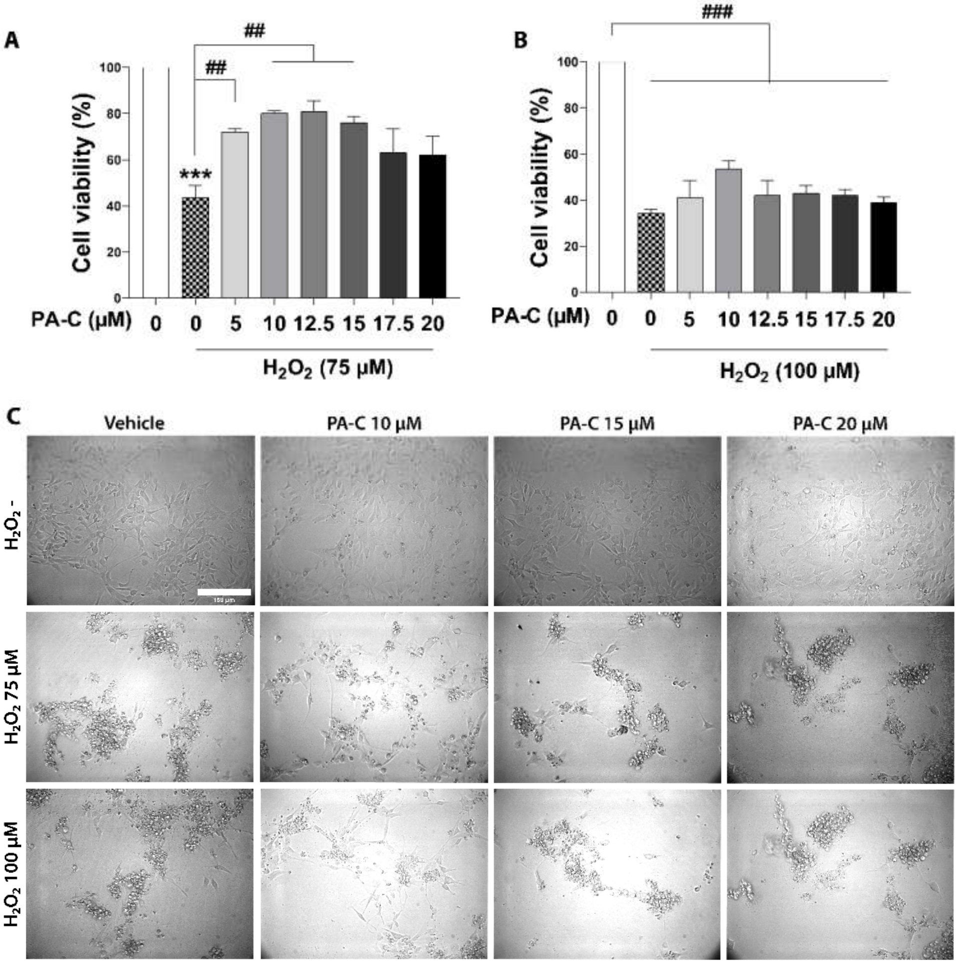

Figure S1. PA-C Protects Against H_2O_2-induced Cytotoxicity in iPSC-NSC. A and B. MTS viability assay analysis. iPSC-NSC were pre-treated with increasing concentrations of PA-C (5, 10, 12.5, 15, 17.5, and 20 µM) for 30 min and then incubated with 75 µM H_2O_2 (**A**) or 100 µM H_2O_2 (**B**) for 24 h. **C.** Representative bright-field images of the iNSC morphology after pre-treatment and cytotoxicity induction with H_2O_2 (scale bar = 150 µm). Data expressed as mean ± S.E.M. (of three independent experiments) determined by one-way ANOVA with Tukey's multiple comparison test. $^{***}p<0.001$ vs control; $^{\#\#}p<0.01$, $^{\#\#\#}p<0.001$ as indicated.

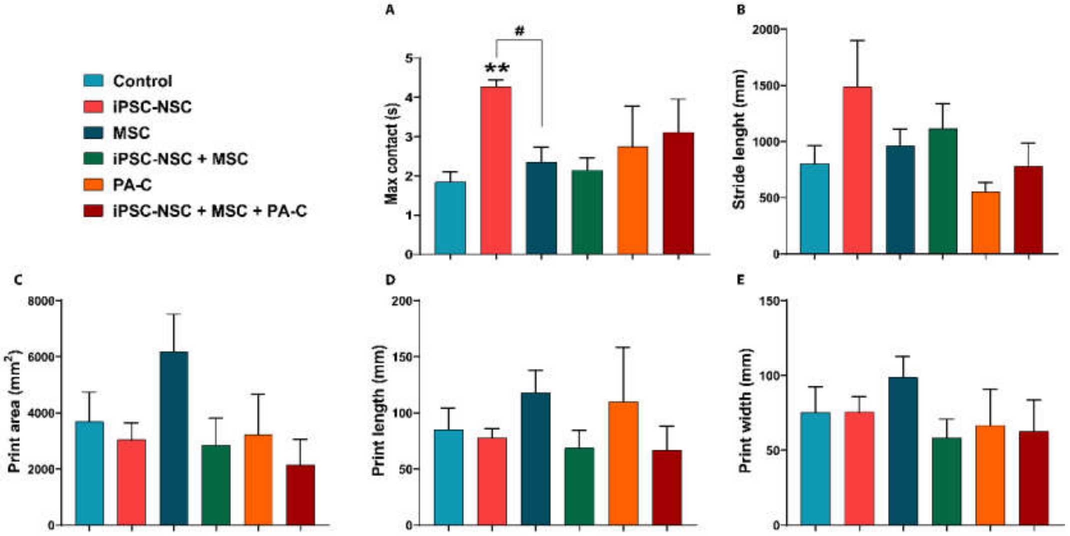

Figure S2. Catwalk Gait Analysis. Quantification of maximum contact (**A**), stride length (**B**), print area (**C**), print length (**D**), and print width (**E**) of the hind limbs for each experimental group. Data are expressed as mean ± S.E.M. determined by one-way ANOVA with Tukey's multiple comparison test (**p<0.01 v control; #p<0.05 as indicated).

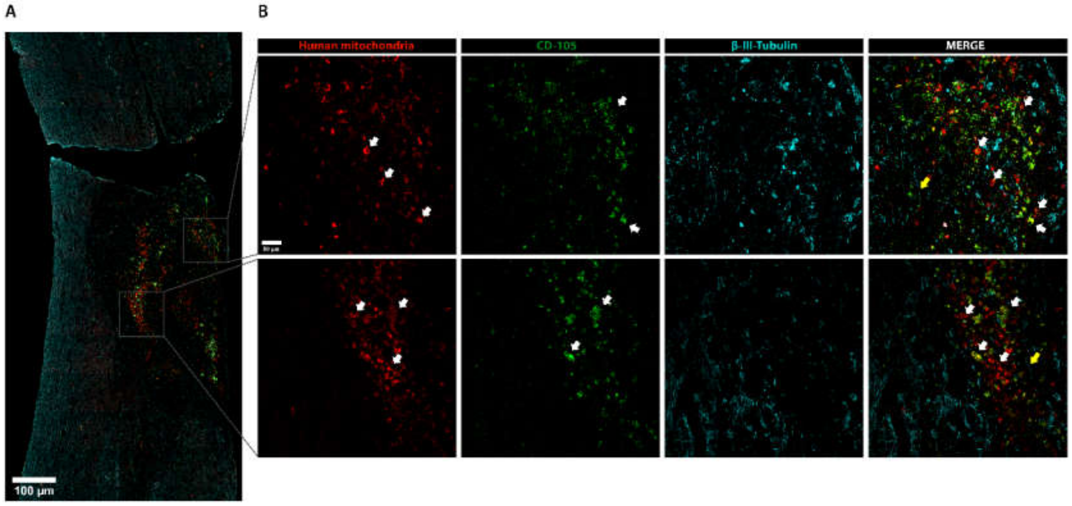

Figure S3. Long Term Survival of Stem Cells Transplants in the Injured Spinal Cord. A. Immunofluorescent longitudinal spinal cord section (including the injury) of ◎-III-Tubulin (blue), human mitochondria (red), and CD-105 (green) at nine weeks post-SCI (scale bar = 150 μm). **B.** Enlarged immunofluorescent images for human mitochondria (red), CD-105 (green), ◎-III-Tubulin (blue), and merge showing the cell grafts eight weeks post-transplantation. White arrows identify human transplants - co-localization of staining for human mitochondria (red) and CD-105 (green) identify MSCs while staining for human mitochondria (red) correspond to iNSC. Yellow arrows indicate ◎-III-Tubulin fibers surrounding cell grafts – staining for human mitochondria (red) alone corresponds to iPSC-NSC .

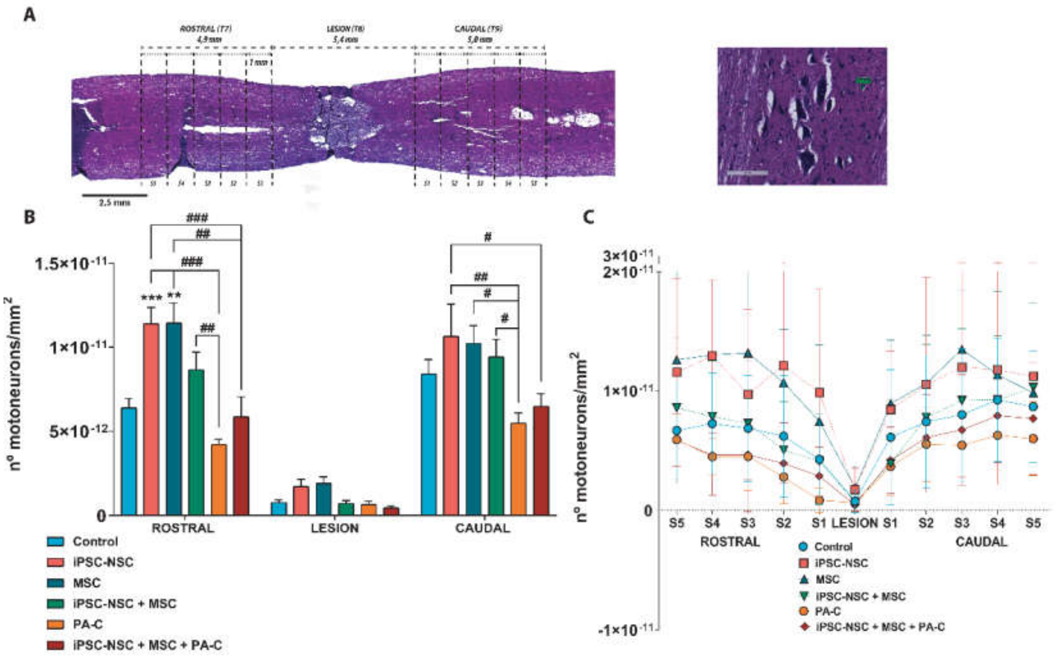

Figure S4. Individual iPSC-NSC and MSC Transplantation Increases Motoneuron Preservation. A. (Left Panel) H/E staining of a longitudinal spinal cord section (including the injury) showing a scheme of the thoracic spinal sections (left panel; scale bar = 2.5 mm). We considered T8 as the epicenter of the lesion and the adjacent sections, both rostral and caudal, as T7 and T9, respectively. Moreover, we considered motoneurons as polygonal cells with a diameter of ≥20 µm (right panel; scale bar = 200 µm). **B.** Quantitative analysis of the motoneuron preservation in the rostral site (T7), lesion (T8), and caudal site (T9). Data (represented as MN/mm^2) expressed as mean ± S.E.M. determined by two-way repeated-measures ANOVA test (Control n=7; iPSC-NSC n=3; MSC n=3; iPSC-NSC+MSC n=7; PA-C n=4; iPSC-NSC+MSC+PA-C n=4). **C.** Overall distribution of motoneurons in the subsections (1 mm) along the spinal cord (represented as MN/mm^2). $^{**}p<0.01$, $^{***}p<0.001$ vs. control; $^{\#}p<0.05$, $^{\#\#}p<0.01$, $^{\#\#\#}p<0.001$ as indicated.

Table S1. *In vitro* effects of 24 h 10 µM PA-C treatment on genes involved in neurogenesis. cell cycle. or apoptosis in iPSC-NSC (entire data set including non-significant changes). Gene profiling analysis was performed with the RT2 Profiler PCR Array. and statistical analysis was performed with the RT2 Profiler RT-PCR Array Data Analysis program. Between-group (treatment vs. control) gene expression differences were evaluated using Student's t-tests from three independent experiments. $^{*}p<0.05$ vs. control.

Gene	2^-ΔCt (PA-C)	2^-ΔCt (Control)	Fold Change PA-C /Control	Student's t-test p-value
ACHE	0.00192772	0.002428775	0.793700526	0.658533174
ADORA1	0.000409962	0.000911867	0.449585268	0.352218187
ALK	0.023000002	0.018170952	1.265756594	0.579864627
APBB1	0.050922408	0.092854209	0.54841249	0.106907649
APOE	0.068130492	0.076121323	0.895025071	0.688202674

APP	0.223928663	0.235605389	0.950439478	0.622268867
ARTN	0.001298544	0.001021174	1.271619166	0.865172281
ASCL1	0.019030331	0.010080967	1.887748625	0.533465017
BDNF	0.01641446	0.009919234	1.654811245	0.460038503
BMP8B	0.030701319	0.073189362	0.419477887	0.262706729
CDK5R1	0.003802361	0.004327585	0.878633452	0.488463785
CDK5RAP2	0.020680924	0.018087178	1.143402487	0.533871905
CHRM2	0.040510633	0.035266555	1.148698355	0.665921593
CREB1	0.176097269	0.212339466	0.829319546	0.518084902
DCX	0.00132277	0.006790708	0.194791145	0.348093365
DLG4	0.009340903	0.02006898	0.465439858	0.126582525
DLL1	0.156884832	0.196297147	0.79922115	0.467585629
DRD2	**0.016490487**	**0.068604377**	**0.240370763**	***0.049718167**
DVL3	0.003882256	0.005254531	0.73883972	0.653697493
EFNB1	0.001109745	0.00867519	0.127921736	0.348145231
EP300	0.171678083	0.361260421	0.475219739	0.086259627
ERBB2	0.030418886	0.012848803	2.367448977	0.779168253
FGF2	0.681968864	0.473393273	1.440596862	0.965849939
FLNA	0.069884248	0.076650789	0.911722489	0.576845193
GDNF	0.675695169	0.56950396	1.186462635	0.703897519
GPI	0.043619321	0.013518802	3.226567037	0.598364595
GRIN1	0.038860397	0.095024573	0.408951029	0.216262202
HDAC4	1.095136811	1.694795392	0.646176415	0.240166645
HES1	0.003724109	0.003153372	1.180992661	0.476145939
HEY1	0.008019777	0.011367912	0.705474904	0.489747437
HEYL	0.000495479	0.00080677	0.614151575	0.389667921
MAP2	0.125966421	0.178143421	0.707106781	0.077775819
MDK	0.114846419	0.073868911	1.554732811	0.749561769
MEF2C	0.008496657	0.003882256	2.188587403	0.651603344

KMT2A	0.000713785	0.000430344	1.658639092	0.903462276
NEUROD1	0.138163117	0.042133486	3.279175994	0.379843798
NEUROG1	2.101050897	3.274128878	0.641712949	0.401714671
NF1	0.180630211	0.201350056	0.897095409	0.565662024
NOG	0.223411875	0.222381875	1.004631674	0.754971384
NOTCH1	0.239446919	0.301684214	0.793700526	0.519147421
NOTCH2	0.011553265	0.015745803	0.733736182	0.550108561
NR2E3	0.002178843	0.001836419	1.186462635	0.433930202
NRCAM	0.014355802	0.022012159	0.652176035	0.343705044
NRG1	0.215801647	0.106169733	2.032609864	0.536412342
NRP1	0.00051652	0.003793586	0.136156082	0.359897344
NRP2	0.17128188	0.077900574	2.198724227	0.923509888
NTF3	0.091575857	0.137844261	0.664342907	0.439514693
NTN1	0.805400224	0.796149413	1.01161944	0.850833706
TENM1	0.178143421	0.078442416	2.271008858	0.410433009
OLIG2	0.013301915	0.02006898	0.662809721	0.491608264
PARD3	0.704389078	0.618899807	1.138131035	0.822883605
PAX3	0.058224808	0.064903696	0.897095409	0.626583097
PAX5	0.02186011	0.033210374	0.65823136	0.570348282
PAX6	0.214806732	0.039676936	5.413894097	0.716219398
POU4F1	0.013487603	0.008360343	1.613283518	0.711368252
PTN	0.257226487	0.033210374	7.745365568	0.780513573
RAC1	0.00356416	0.002621202	1.359742373	0.474356553
RTN4	0.581470448	0.481111918	1.208597056	0.806057704
S100A6	0.44476375	0.34814924	1.277508892	0.964490187
S100B	3.184597652	3.214166008	0.990800613	0.906197452
SHH	0.002276623	0.008939714	0.254663952	0.399013848
SLIT2	0.904031185	0.206533032	4.377174805	0.712670311
SOD1	0.010655763	0.01800379	0.591862244	0.344690673

SOX2	0.000518912	0.000597452	0.868541486	0.747178997
SOX8	0.68989296	0.470123307	1.467472363	0.621816567
STAT3	0.022165265	0.017310339	1.280463977	0.476661094
TGFB1	0.518031277	0.518031277	1	0.806155891
TH	0.000818032	0.00085672	0.954841604	0.721578193
TNR	0.095464696	0.142047411	0.6720622	0.68573148
VEGFA	0.883383084	0.770808531	1.146047362	0.841497866
